# Enlarged Pore Size Chiral Mesoporous Silica Nanoparticles Loaded Poorly Water-Soluble Drug Perform Superior Delivery Effect

**DOI:** 10.3390/molecules24193552

**Published:** 2019-09-30

**Authors:** Yingyu Guo, Kaijun Gou, Baixue Yang, Yumei Wang, Xueyu Pu, Sanming Li, Heran Li

**Affiliations:** 1 School of Pharmacy, Shenyang Pharmaceutical University, Shenyang 110016, China; 18524446201@163.com (Y.G.); kaijungou@126.com (K.G.); hebtuybx@163.com (B.Y.); 18341460263@163.com (Y.W.); 18841480115@163.com (X.P.); 2 School of Pharmacy, China Medical University, Shenyang 110122, China

**Keywords:** enlarged mesopores, chiral mesoporous silica nanoparticles, drug delivery

## Abstract

Large mesopores of chiral silica nanoparticles applied as drug carrier are worth studying. In this study, chiral mesoporous silica nanoparticles (CMSN) and enlarged chiral mesoporous silica nanoparticles (E-CMSN) with a particle size from 200 to 300 nm were synthesized. Fourier transform infrared spectrometer (FTIR), circular dichroism spectrum, scanning electron microscopy (SEM), transmission electron microscope (TEM), and nitrogen adsorption/desorption measurement were adopted to explore their characteristics. The results showed that the surface area, pore volume, and pore diameter of E-CMSN were higher than those of CMSN due to enlarged mesopores. Poorly water-soluble drug nimesulide (NMS) was taken as the model drug and loaded into carriers using adsorption method. After NMS was loaded into CMSN and E-CMSN, most crystalline NMS converted to amorphous phase and E-CMSN was superior. The anti-inflammatory pharmacodynamics and in vivo pharmacokinetics results were consistent with the wetting property and in vitro drug dissolution results, verifying that NMS/E-CMSN exhibited superior NMS delivery system based on its higher oral relative bioavailability and anti-inflammatory effect because its enlarge mesopores contributed to load and release more amorphous NMS. The minor variations in the synthesis process contributed to optimize the chiral nano-silica drug delivery system.

## 1. Introduction

In recent years, mesoporous silica nanoparticles (MSN) fabricated based on various kinds of templates have aroused a lot of interest owing to their rigid frame, good biocompatibility and biodegradability, unique structural features (tunable mesopores in the range of 2–50 nm, large pore volume as well as total surface area), and facile surface modification [1,2,3,4]. To better play the bio-recognition performance, MSN will be modified by chiral small molecules [5,6,7,8], whereas the chiral mesoporous silica nanoparticles (CMSN) will tend to reduce the mesopores size. Enlarging the mesopores of CMSN can load biomacromolecule and protect its activity [9]. There are many methods to realize CMSN with large mesopores, including prolonging the length of the template agent chain, changing templates, adjusting pH of the synthesized system and adding additives. For instance, the mesopores can be enlarged to 10 nm using designed surfactant [10,11,12]. It was reported that mesopores in the range of 3.8 to 20 nm can be regulated by controlling systemic pH. However, the pH cannot be precisely controlled in the sol to gel preparing process, and the mesoporous range was a little wide [13]. In addition, 1,3,5-trimethylbenzene (TMB) and 1,3,5-triisopropylbenzene can be used to enlarge mesopores of MSN [14,15]. The pore size of MCM-41 increased up to 12 nm with rising amounts of mesitylene. MCM-41 synthesized with 1,3,5-triisopropylbenzene as an auxiliary chemical displayed large mesopores, whereas their pore sizes were not more than 4.0 nm [16]. The pore size of MCM-41 prepared with a 3:1 mixture of 1,3,5-triisopropylbenzene and mesitylene reached 4.7 nm [17].

It is widely accepted that CMSN can be applied as drug carrier to establish a profound of drug delivery systems, primarily covering controlled release, stimuli-response release and multifunctional release [8,18,19]. One outstanding advantage of CMSN is to enhance the dissolution and bioavailability of poorly water-soluble drugs [20]. In the global drug market, numerous drug candidates, especially BCS II drugs [21], exhibit poor water solubility, and these drugs cannot exert therapeutic effects due to the low drug concentration in the site of absorption, which significantly limits their application. The poor water solubility is correlated with stable crystals forms of drug, and therefore it is urgent to overcome the forces attaching the drug molecule within the crystalline lattice. It is generally known that the BCS classifies drugs refer to oral administration into four groups on the basis of aqueous solubility/dissolution, as well as the intestinal epithelium permeability. Drug belongs to class I when its permeability and solubility are both high. If its permeability is high while solubility is low, it will belong to class II. Class III drug exhibits low permeability but high solubility. Those drugs with both low solubility and low permeability belong to class IV [22]. In existing research, CMSN has performed meritorious service for improving the dissolution and bioavailability of BCS II drugs since the mesoporous space limits the structure of crystal drugs and converts its crystalline phase to amorphous state [23,24].

Though great efforts have been made to study the synthesis process and application of CMSN with enlarged pore size, the potential ability of its drug delivery effects should be systematically elucidated [25,26]. In this study, E-CMSN was synthesized with TMB as pore-enlarging agent. Fourier transform infrared spectrometer (FTIR), circular dichroism spectrum, scanning electron microscopy (SEM), transmission electron microscope (TEM), and nitrogen adsorption/desorption measurement were applied to characterize E-CMSN with CMSN as the control sample. Poorly water-soluble drug nimesulide (NMS), known as a selective nonsteroidal anti-inflammatory drug [27], was chosen as the model drug. The LogP value of NMS is 3.79. NMS belongs to semiselective COX-2 inhibitors, displaying pharmacological activities of pain relieving, painful osteoarthritis extra-articular disorders and other acute pain states. NMS was loaded into CMSN and E-CMSN with high drug loading capacity, respectively. The crystalline state of NMS in these two carriers was studied using differential scanning calorimeter (DSC) and X-ray power diffraction (XRD). Drug delivery performances of CMSN and E-CMSN in delivering NMS were studied based on dissolution test, contact angle measurement, in vivo pharmacodynamics, anti-inflammatory pharmacodynamics and mucous membrane adhesion studies.

## 2. Results and Discussions

### 2.1. Characterization

**Figure 1:**
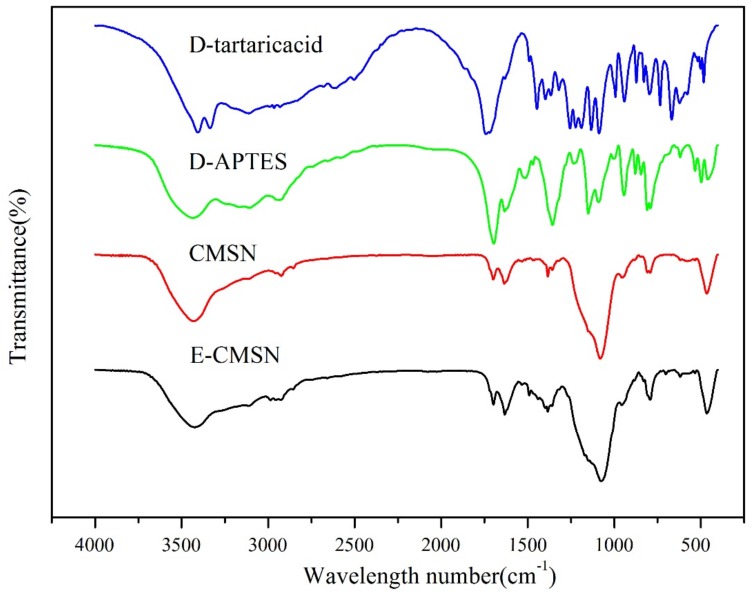
FTIR spectra of D-tartaric acid, D-APTES, chiral mesoporous silica nanoparticles (CMSN) and enlarged chiral mesoporous silica nanoparticles (E-CMSN) from 400 to 4000 cm^−1^.

**Figure 2:**
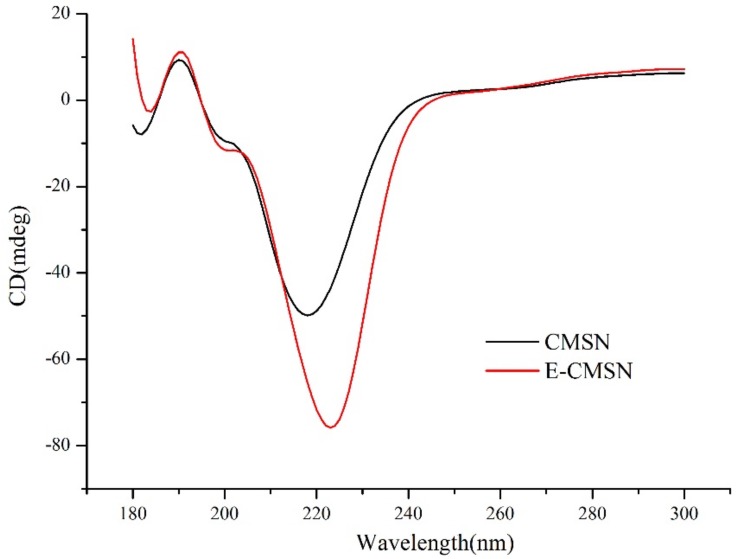
Circular dichroism spectra of CMSN and E-CMSN from 180 to 300 nm.

As shown in Figure 1, both CMSN and E-CMSN displayed crucial peaks belonging to silica, covering bending vibration peak of Si-O-Si at 456.0 cm^−1^ and 465.7 cm^−1^, Si-O-Si symmetric stretching vibration at 798.4 cm^−1^ and 792.9 cm^−1^, asymmetric stretching vibration absorption peak of Si-O-Si at 1080.9 cm^−1^ and 1075.6 cm^−1^, OH of Si-OH antisymmetric stretching vibration at 3425.2 cm^−1^ and 3425.5 cm^−1^, suggesting that the silica frame was successfully formed. Furthermore, N-H deformation vibration at 1635.2 cm^−1^ and carbonyl stretching band at 1699.2 cm^−1^ that shown in spectrum of CMSN originated from the chiral groups that grafted onto the silica frame [7]. After mesopores were enlarged, the two peaks were also seen with a little lower shift than CMSN (N-H deformation vibration at 1632.4 cm^−1^ and carbonyl stretching band at 1698.7 cm^−1^ from the spectrum of E-CMSN), implying that the existence state of functional groups in E-CMSN can slightly different from CMSN for the pore enlargement management. Circular dichroism spectrum is a critical parameter to confirm the chiral property. As shown in Figure 2, the reversal peak around 220 nm of CMSN and E-CMSN was ascribed to the corresponding D-tartaric acid [28], thus confirming the chirality of CMSN and E-CMSN. After pore enlargement, the chirality of E-CMSN became stronger evidenced by its stronger CD signal, suggesting that the enlarged pores of CMSN enhanced chirality.

**Figure 3:**
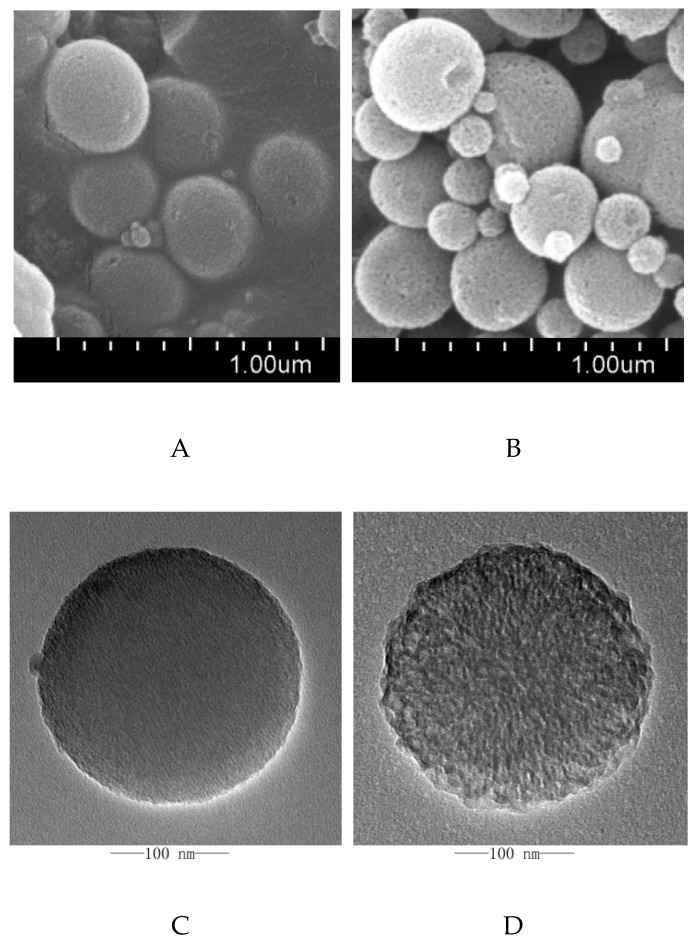

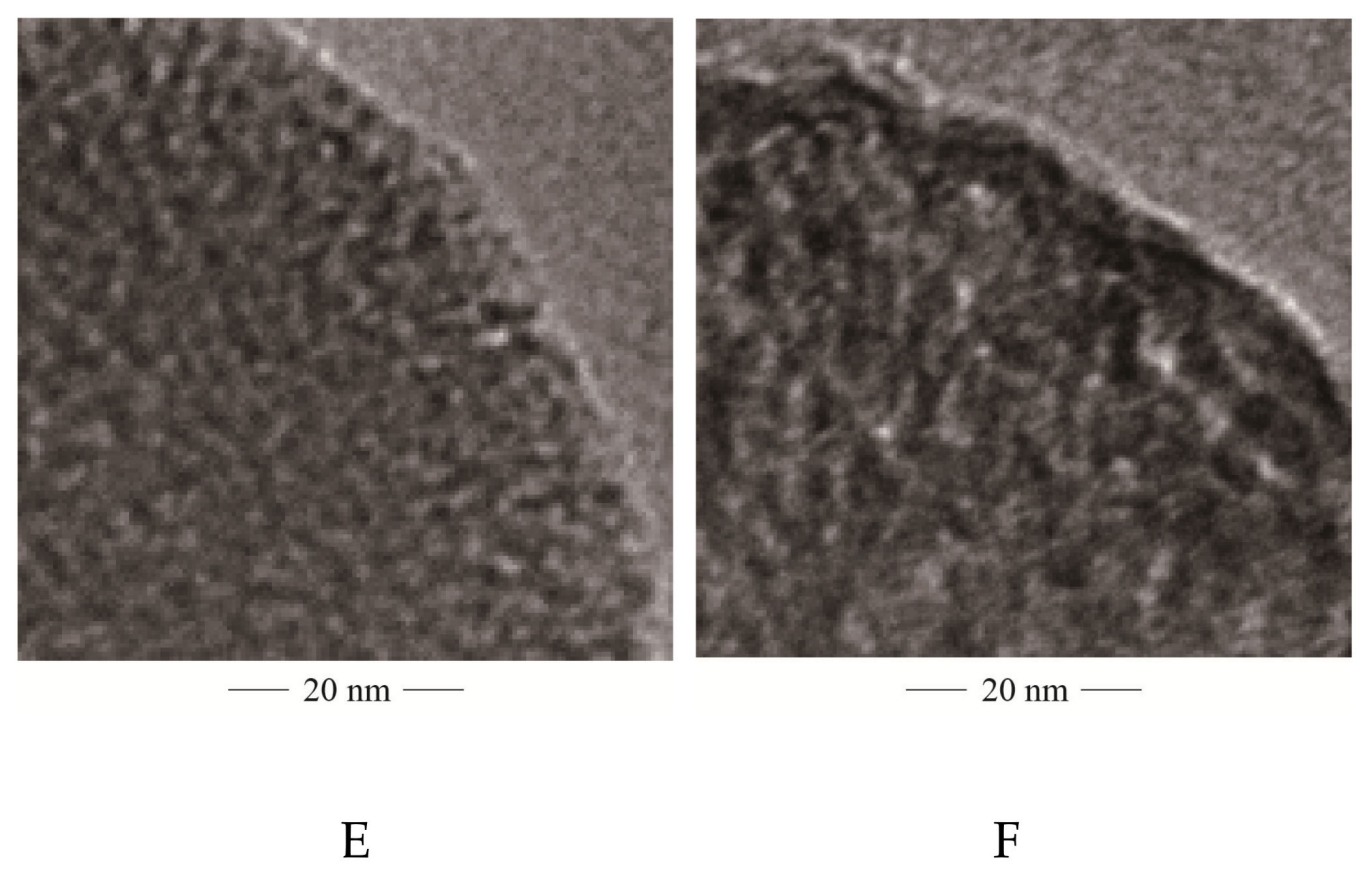
(**A**), SEM images of CMSN; (**B**), SEM images of E-CMSN; (**C**), TEM images of CMSN; (**D**), TEM images of E-CMSN; (**E**), Enlarged TEM images of CMSN; (**F**), Enlarged TEM images of E-CMSN.

**Figure 4:**
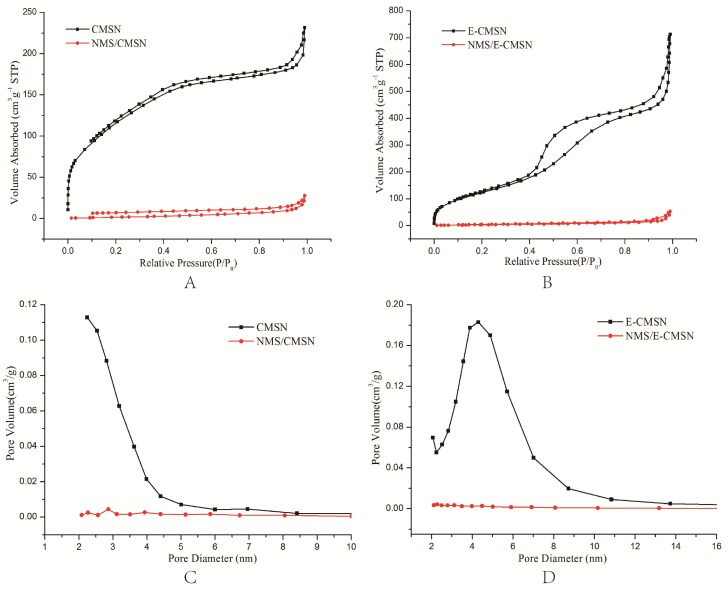
(**A**,**B**), Nitrogen adsorption/desorption isotherm of CMSN, NMS/CMSN, E-CMSN, and NMS/E-CMSN; (**C**,**D**), Pore size distribution of CMSN, NMS/CMSN, E-CMSN, and NMS/E-CMSN.

CMSN and E-CMSN morphology were studied using SEM and TEM, and the images were shown in Figure 3. Visually, both CMSN and E-CMSN were nanoparticles with particle size in the range of 200 to 300 nm. Their surface was rough due to the porous channels inside the particles and functionalized groups onto the silica frame. CMSN was shaped like a ball of wool. After being enlarged pores, it turned out to be an incompact ball of wool with obvious larger pores observed from the morphology surface and transmitting porous channels frame. The result revealed that the pore enlarging process occurred through the whole silica frame establishment process, and that enlarged pores were significant obvious. Nitrogen adsorption/desorption isotherms and pore size distribution curves of CMSN and E-CMSN were shown in Figure 4. The nitrogen adsorption/desorption isotherms of CMSN showed a hysteresis loop in the relative pressure of 0.2 to 1.0, while the hysteresis loop of E-CMSN displayed in the relative pressure of 0.4 to 1.0. Obviously, the hysteresis loop of E-CMSN was larger than that of CMSN. The enlarged hysteresis loop contributed to large surface area (CMSN: 440.666 cm^2^/g; E-CMSN: 474.360 cm^2^/g) and pore volume (CMSN: 0.358 cm^3^/g; E-CMSN: 1.103 cm^3^/g), revealing that the pore enlarging effect of E-CMSN is effective. Furthermore, pore diameter intuitively reflected the pore enlarging phenomenon since the pore diameters of CMSN and E-CMSN were 2.2 nm and 4.3 nm. The pore enlargement effect of E-CMSN was obvious since most common pore diameter of mesoporous silica nanoparticles was in the range of 2 to 3.5 nm [28,29].

### 2.2. Drug Loading and Properties

The drug loading capacity of NMS/CMSN and NMS/E-CMSN were 24.51% ± 0.27% and 27.45% ± 0.19% respectively. The loading capacity of NMS/E-CMSN was higher than that of NMS/CMSN even though their differences were not significant, illustrating that the enlarged pores of E-CMSN exhibited potential ability to entrap more NMS molecules. After drug loading, the drug crystalline state property was studied using BET, XRD, and DSC measurements. According to the BET result (Table 1), all the parameters (surface area, pore volume, as well as and pore diameter) were significantly decreased after loading NMS into the two carriers. The reduced values of these parameters calculated from E-CMSN and NMS/E-CMSN (narrowed surface area: 459.725 cm^2^/g; decreased pore volume: 1.021 cm^3^/g) were higher than those from CMSN and NMS/CMSN (narrowed surface area: 431.258 cm^2^/g; decreased pore volume: 0.315 cm^3^/g), further confirming the superior drug loading capacity of E-CMSN.

**Figure 5:**
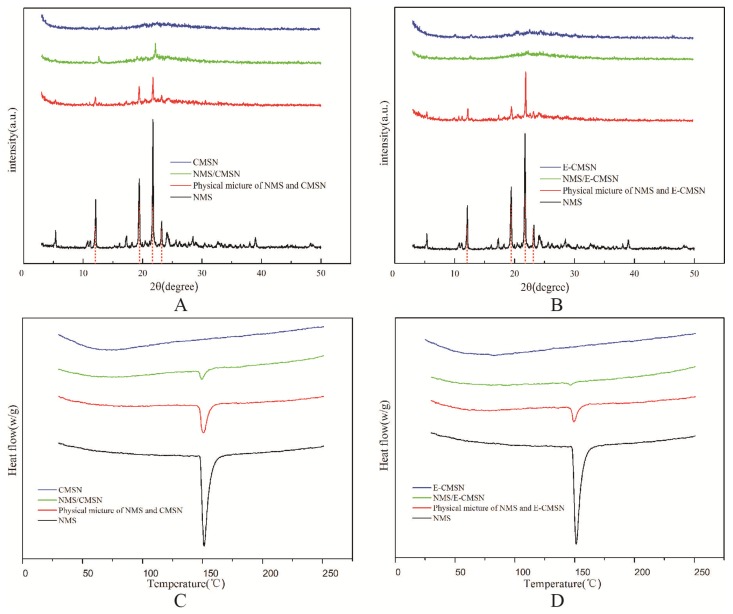
(**A**), XRD patterns of NMS, CMSN, NMS/CMSN, physical mixture of NMS and CMSN (1:3, *w*/*w*); (**B**), XRD patterns of NMS, E-CMSN, NMS/E-CMSN, physical mixture of NMS and E-CMSN (1:3, *w*/*w*); (**C**), DSC curves of NMS, CMSN, NMS/CMSN, physical mixture of NMS and CMSN (1:3, *w*/*w*); (**D**), DSC curves of NMS, E-CMSN, NMS/E-CMSN, physical mixture of NMS and E-CMSN (1:3, *w*/*w*).

XRD and DSC measurements explained the drug crystalline state after being loaded into the two carriers [30,31]. As shown in Figure 5, NMS showed its melting peak at 147 °C owing to its crystalline nature. The diffraction pattern of pure NMS was also highly crystalline, and its XRD pattern had obvious diffraction peaks at 12.12°, 19.44°, 21.75°, and 23.22°. These were characteristic diffraction peaks of NMS [32]. The physical mixture of the carrier and NMS also exhibited the characteristic diffraction peaks of NMS with a lower peak intensity than the pure drug. After loading NMS into the carrier, CMSN was able to convert most drug crystalline state to amorphous phase. It was evidenced by the fact that the drug diffraction peaks were difficult to be observed, and the drug melting peak became quite small. It should be noted that E-CMSN performed superior ability to change drug crystalline state because almost no drug diffraction peaks and drug melting peak were shown in the profiles of NMS/E-CMSN, demonstrating that E-CMSN with enlarged pore diameter of 4.3 nm was qualified for loading amorphous NMS, which become another advantage for its application.

### 2.3. Wetting Property and Drug Dissolution

**Figure 6:**
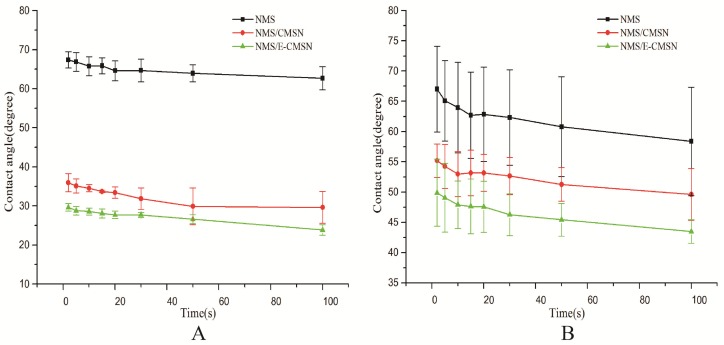
Contact angle measurement results of NMS, NMS/CMSN, NMS/E-CMSN with (**A**), pH 6.8 PBS; (**B**), pH 6.8 PBS (including trysin).

Figure 6 suggested that NMS had poor water wetting property evidenced by its high contact angle at initial moment when contacting with medium and its significant low wetting rate. However, the contact angle of NMS/CMSN and NMS/E-CMSN were extremely lower than NMS, suggesting that the two carriers had functions of improving NMS wetting property [33,34]. Furthermore, E-CMSN performed better ability to enhance NMS wetting property, probably because (1) both CMSN and E-CMSN converted crystalline NMS into amorphous NMS to increase NMS solubility; (2) E-CMSN with enlarged pore diameter of 4.3 nm was qualified for loading amorphous NMS.

**Figure 7:**
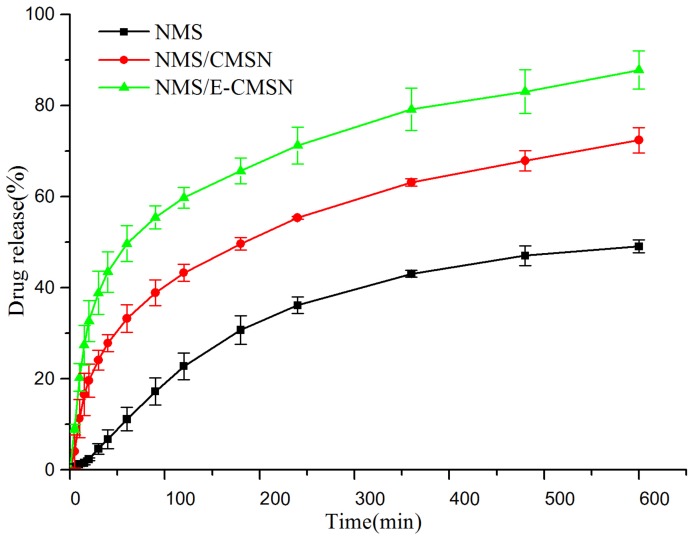
In vitro release profiles of NMS, NMS/CMSN, and NMS/E-CMSN in pH 6.8 PBS at 37 °C.

In vitro dissolution of NMS, NMS/CMSN and NMS/E-CMSN in pH 6.8 PBS medium was shown in Figure 7. Two important points can be concluded based on the in vitro dissolution profile. First, CMSN and E-CMSN could improve NMS dissolution for their significant higher drug dissolution profiles compared to NMS because most loaded NMS was amorphous and drug wetting property had been enhanced [35,36,37]. Second, the in vitro dissolution of NMS/E-CMSN was significantly higher than that of NMS/CMSN, demonstrating that the enlarged mesopores were favorable for amorphous NMS release since the large mesopores provided more space for the entrance of dissolution medium [20]. Another two key points can be summarized according to the results of contact angle measurement and in vitro drug dissolution. The variation of contact angle between crystalline NMS and amorphous NMS was quite large while the contact angle differences from NMS/CMSN and NMS/E-CMSN was small, suggesting that the wetting property of amorphous NMS was good so that their drug dissolution was high.

### 2.4. In Vivo Biological Studies

**Figure 8:**
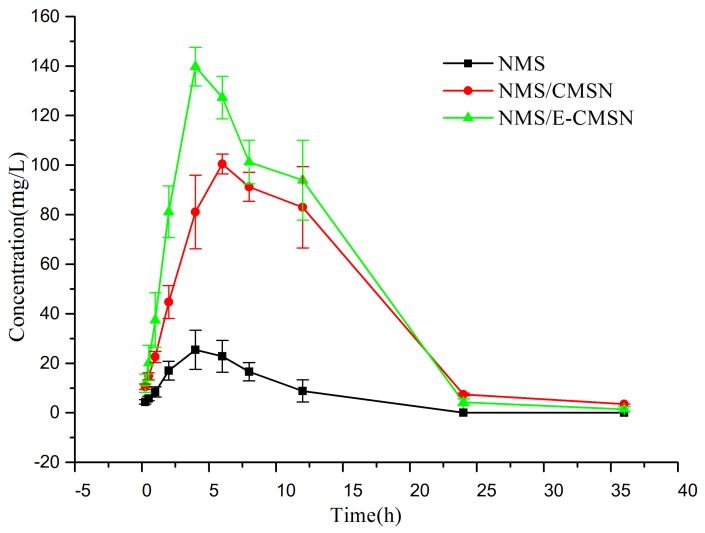
Plasma drug concentration profiles of NMS, NMS/CMSN, and NMS/E-CMSN (n = 3).

The in vivo biological effect of NMS/CMSN and NMS/E-CMSN was studied by in vivo pharmacokinetics, anti-inflammatory pharmacodynamics, and mucous membrane adhesion studies with NMS as the control. Pharmacokinetics profiles and parameters were shown in Figure 8 and Table 2, and anti-inflammatory pharmacodynamics and mucous membrane adhesion results are presented in Figure 8. Both CMSN and E-CMSN enhanced NMS bioavailability evidenced by their prolonged MRT, enhanced C_max_ and AUC. The relative bioavailability of NMS/CMSN and NMS/E-CMSN reached to 765.76% and 905.04%, respectively, thereby enhancing contributed the enhanced NMS dissolution based on the change of crystalline NMS to amorphous NMS using CMSN and E-CMSN. The result also confirmed that E-CMSN with enlarged mesopores was able to perform superior NMS delivery effect owing to its high oral bioavailability [38,39]. The reason was because NMS/E-CMSN presented higher drug dissolution than that of NMS/CMSN as stated in previous discussion, thereby enhancing in vivo NMS absorption. Furthermore, the significantly enhanced relative bioavailability suggested that crystalline state of NMS was its major limitation for adsorption, and therefore the more amorphous NMS in the formation, the higher oral bioavailability.

**Figure 9:**
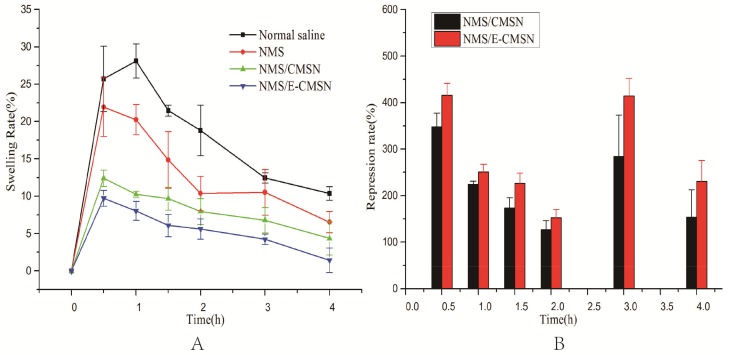
(**A**), the effects of NMS, NMS/CMSN, NMS/E-CMSN on mouse ankle swelling rate; (**B**), the anti-inflammatory repression rate of NMS/CMSN and NMS/E-CMSN.

**Figure 10:**
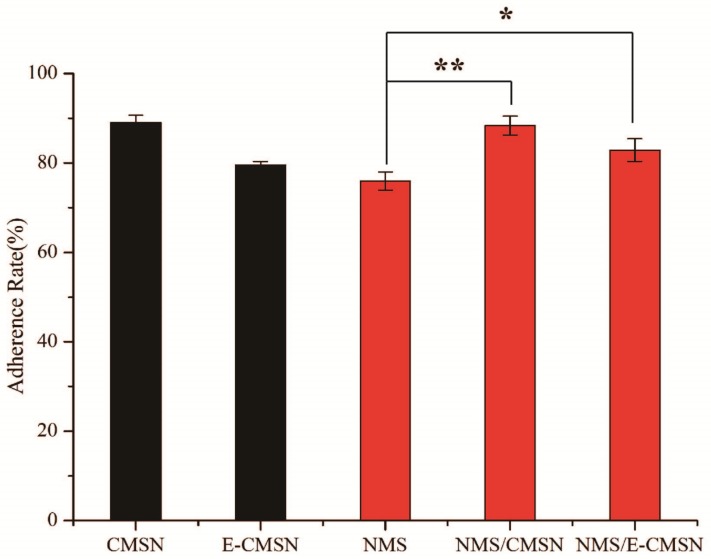
The mucous membrane adhesion of CMSN, E-CMSN, NMS, NMS/CMSN, NMS/E-CMSN. (n = 3, *: *p* < 0.05, **: *p* < 0.01).

The anti-inflammatory pharmacodynamics result was in agreement with in vivo pharmacokinetics result. As can be seen in Figure 9, paw model was successfully built since the paw degree of rats increased gradually along with time when the rats administered normal saline. NMS started to exhibit anti-inflammatory function after 0.5 h, so did NMS/CMSN and NMS/E-CMSN. According to swelling rate profiles, NMS exerted anti-inflammatory pharmacodynamics effect, and both NMS/CMSN and NMS/E-CMSN exerted stronger anti-inflammatory effect than NMS, evidencing that the enhanced oral bioavailability led to better drug effect. NMS/E-CMSN exhibited superior anti-inflammatory pharmacodynamics based on swelling rate profiles and repression rate result because its enlarged mesopores contributed to load more amorphous NMS. In Figure 10, it was noteworthy that the adhesion rates of CMSN and E-CMSN were a little higher than NMS, suggesting that the silicon hydroxyl groups on the surface of carriers had good mucous membrane adhesion ability since hydroxyl groups had strong affinity with hydrophilic proteins on the membrane [40,41]. The adhesion rate of E-CMSN was a litter lower than CMSN as the number of silicon hydroxyl groups on the surface was lower due to enlarged mesopores. After loading NMS, the adhesion rates of NMS/CMSN and NMS/E-CMSN were higher those of NMS due to the good mucous membrane adhesion ability of the two carriers, which improves their application in orally delivering NMS.

## 3. Materials and Methods

### 3.1. Materials

Tetraethoxysilane (TEOS, ≥99%) was purchased from Aladdin (Shanghai, China). Absolute ethyl alcohol was bought from Bodi chemical Co.,Ltd (Tianjing, China). Methyl alcohol was purchased from Hengxing chemical Co.,Ltd (Tianjing, China). Aminopropyl triethoxysilane (APTES) was provided by Xiya chemical Co.,Ltd (Shandong, China). Octadearyl dimethyl ammonium chloride was bought from Beijing coupling technology Co.,Ltd (Beijing, China). Rat pharmacodynamics and anti-inflammatory pharmacodynamics were conducted based on the ethical guidelines that approved by the Ethics Review Committee for Animal Experimentation of Shenyang Pharmaceutical University (Shenyang, China).

### 3.2. Preparation of CMSN and E-CMSN

**Figure 11:**
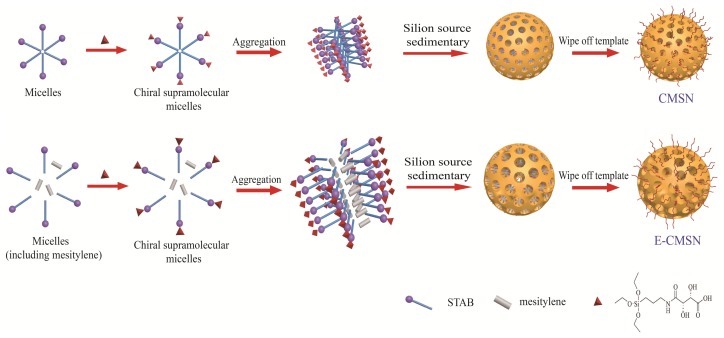
Synthetic routes of CMSN and E-CMSN.

CMSN adopted as control carrier was synthesized as follows. 6.6 mg/mL Octadecy trimethyl ammonium bromide (STAB) was prepared using double distilled water and anhydrous ethanol at ambient temperature. In the meantime, 8.547 mmol APTES was added into 15 mg/mL D-tartaric acid ethanol, and the obtained white precipitate (D-APTES) was collected. Subsequently, 68.517 mmol ammonia liquor and 0.8 g D-APTES were added to the template solution under stirring. 17.913 mmol TEOS was dropped into the homogeneous solution under vigorous stirring. After 4 h, the obtained mixture was left statically for 1 day. Lastly, the mixture was washed by water and ethanol and then dried in oven. The template was removed by refluxing in hydrochloric acid methanol solution to get CMSN. E-CMSN was prepared using the above synthesized process except that 488 μL TMB was added into template solution before the adding of ammonia liquor. The schematic diagram of synthetic routes was shown in Figure 11.

### 3.3. Characterization

#### 3.3.1. FTIR

FTIR (Spectrum 1000, PerkinElmer, USA) spectra of CMSN and E-CMSN were measured from the spectral region 500 to 4000 cm^−1^. Samples were produced by grounding with KBr gently and respectively [42].

#### 3.3.2. Circular Dichroism Spectrum

3mg processed carrier was accurately weighed and mixed with 22 mg KBr uniformly, then the mixture was compressed into tablet using infrared tablet press. Circular dichroism apparatus (MOS-500, Bio-Logic, France) was employed to confirm chirality of CMSN and E-CMSN [43].

#### 3.3.3. SEM

SEM was obtained with SU8010 (Hitachi, Japan) to characterize the morphology of CMSN and E-CMSN. Before measurement, samples were mounted onto metal stubs using double-sided adhesive tape and then sputtered with a thin layer of gold under vacuum.

#### 3.3.4. TEM

The porous structure of CMSNs and E-CMSN was tested using JEM2100 TEM instrument (JEOL. Japan). Sample ethanol solution was withdrawn and then displayed onto porous carbon films.

#### 3.3.5. Nitrogen Adsorption/Desorption Measurement

Specific porous structure of CMSN and E-CMSN was studied using V-Sorb 2800P (Beijing, China). The major parameters, covering specific surface area (S_BET_), total pore volume (V_t_) and pore size distributions, were obtained according to nitrogen adsorption in the relative pressure range from 0.05 to 0.2, the amount adsorbed at a relative pressure of 0.99, and adsorption branches of isotherms respectively.

### 3.4. Drug Loading Procedure

The solvent drying method was used to load model drug NMS into CMSN or E-CMSN. Firstly, 2mL NMS acetone solution (10 mg/mL) was mixed with 60 mg CMSN or E-CMSN. The system was sealed and stirred gently for 1 day at room temperature. Then the lid was opened with stirring until solvent was evaporated completely to get drug loaded carrier. Finally, unloaded NMS was washed using distilled water [28]. Drug loading capacity was calculated by taking an accurately weighed quantity of NMS loaded carrier, then extracting the loaded NMS completely using 0.05 mol/L NaOH from CMSN or E-CMSN and analyzed drug content with UV-1750 (Shimadzu, Japan) at 393 nm.

Drug loading capacity (%) = (W drug in nanoparticles/W nanoparticles) × 100(1)

### 3.5. XRD

XRD of a range of samples, covering NMS, CMSN, physical mixture of NMS and CMSN, NMS loaded MSN (NMS/CMSN), E-CMSN, physical mixture of NMS and E-CMSN, as well as NMS loaded E-CMSN (NMS/CMSN), were analyzed using a EMPYREAN XRD (PANalytical B.V., Netherlands). The wavelength was scanned between 5 and 40 °C 2θ with a 0.04° step size.

### 3.6. DSC

DSC thermograms (HCT-1, Beijing, China) of a range of samples, covering NMS, CMSN, physical mixture of NMS and CMSN, NMS/CMSN, E-CMSN, physical mixture of NMS and E-CMSN, as well as NMS/E-CMSN, were obtained at heating temperature from 30 °C to 500 °C at the rate of 10 °C/min.

### 3.7. In Vitro Drug Dissolution

NMS, NMS/CMSN, and NMS/E-CMSN in vitro dissolution were performed in 250 mL dissolution medium (pH 6.8 phosphate buffer solution). The experiment was performed at 37 °C, 50 rpm and time was recorded at the beginning of the experiment. Aliquots (5 mL) were withdrawn at appropriate time intervals and replaced with 5 mL of fresh dissolution medium after each sampling to maintain constant volume [44]. The sample medium was analyzed using UV-1750 (Shimadzu, Japan) at the wavelength of 393 nm after going through 0.22 μm microporous membrane.

### 3.8. Contact Angle Measurement

To study the wettability differences after loading NMS into the two carriers, dynamic contact angle measurement was performed using JCY series (Shanghai, China). The working conditions covered: Automatic contact angle meter model, pressurized tablet of sample, as well as a drop of dissolution medium (2 μL). The measurement was started when dissolution medium dropped onto the tablet [45,46].

### 3.9. In Vivo Pharmacokinetics

9 Male Wistar rats weighing 200 ± 20 g were randomly split into three groups (A group, NMS; B group, NMS/CMSN; C group, NMS/E-CMSN) and fasted for 12 h but given free access to water prior to the experiment. The three samples were suspended in 1.5 mL normal saline with the drug dose of 40 mg/kg and then orally administered (intragastric administration) to rats. Blood media (0.5 mL) of each animal was collected via the suborbital vein at different time intervals after administration and immediately centrifuged at 8000 rpm for 10 min to collect plasma. Subsequently, the obtained plasma was then stored at 20 °C until analysis. 200 μL plasma sample was mixed with 80 μL of an internal standard solution (0.5 mg/mL) and 400 μL acetonitrile. The mixture was vortexed for 2 min. After centrifugation at 10,000 rpm for 5 min, the supernatant of each sample (20 μL) was subjected to HPLC analysis [47]. The working conditions for HPLC analysis included: Mobile phase: methanol: pH 7.3 potassium dihydrogen phosphate buffer (65:35, *v*/*v*); Column temperature: 30 °C; Analysis wavelength: 370 nm; Flow rate: 1.0 mL/min.

### 3.10. Anti-Inflammatory Pharmacodynamics

Right hind ankle perimeter of rats was ascertained to study the anti-inflammatory biological effect of NMS/CMSNs or NMS/E-CMSN. Twelve male Sprague–Dawley rats (200 ± 20 g) were separated into four groups (Group A: Normal saline; Group B: NMS; Group C: NMS/CMSN; Group D: NMS/E-CMSN) and then fasted overnight before experiment. The rats in Group B, Group C and Group D were administered orally with corresponding nanoparticles containing 40 mg/kg NMS, while the rats in Group A were administered orally with normal saline. Afterwards, the perimeters of right hind ankles were ascertained. After half an hour, 0.1 mL 1% carrageenan solution was injected subcutaneously into the paw of each rat. The perimeters of right hind paw ankle after injection were measured at predetermined time intervals (0.5 h, 1 h, 1.5 h, 2 h, 3 h, and 4 h). Then, the following parameters were calculated [48].

(2) Repressionrate(%)=(Ct−C0)negtive−(Ct−C0)test(Ct−C0)negtive−(Ct−C0)positive

(3) $$Swelling\;rate(\%)=\frac{C_{t}-C_{0}}{C_{0}}\times 100$$

where C_0_ denotes original perimeter of right hind ankle; C_t_ is the measured perimeter of right hind ankle.

### 3.11. Mucous Membrane Adhesion

Healthy Kunming mice were sacrificed after being fastened for 8 h. Then, the small intestine was removed, and the mice were divided into 3–4 segments (4 cm per segment). The inside food residue was rinsed with normal saline. The gel layer was kept on the small intestine and then used in a flat state [49,50]. Ep tubes (10 mL) were prepared for each sample and weighed in advance. 20 mg carrier (CMSN, E-CMSN), 5 mg NMS, and NMS/CMSN containing 5 mg NMS as well as NMS/E-CMSN containing 5 mg NMS were spread evenly on the surface of the mucosa, respectively, and then wet gently. Next, the glass plate was tilted to the horizontal plane. At a 45° angle, the intestinal fluid (without enzyme) was washed at 37 °C at a certain flow rate (2 mL/min), and the rinse solution was collected for 5 min. After centrifuging, the supernatant liquid was discarded, and the lower layer carrier was dried and then weighed to calculate the adhesion amount. The ep tube containing the drug carrier rinsing solution was sonicated for 20 min and then diluted with a simulated intestinal fluid. Lastly, the absorbance was measured to ascertain the eluted drug content and the adhesion rate.

## 4. Conclusions

In this study, CMSN and E-CMSN with a particle size from 200 to 300 nm were synthesized. The surface area (CMSN: 440.666 cm^2^/g; E-CMSN: 474.360 cm^2^/g), pore volume (CMSN: 0.358 cm^3^/g; E-CMSN: 1.103 cm^3^/g) and pore diameter (CMSN: 2.2 nm; E-CMSN: 4.3 nm) of E-CMSN were higher than those of CMSN as mesopores were enlarged. After NMS was loaded into CMSN and E-CMSN, most crystalline NMS converted to amorphous phase and E-CMSN was superior, as confirmed by XRD and DSC analysis. The wetting property of NMS and its dissolution were significantly enhanced by the contribution of the two carriers. The anti-inflammatory pharmacodynamics and in vivo pharmacokinetics results were consistent with the wetting property and in vitro drug dissolution results, proving that NMS/E-CMSN exhibited superior NMS delivery system based on its higher oral relative bioavailability and anti-inflammatory effect since its enlarge mesopores contributed to load more amorphous NMS. It is considered that E-CMSN with superior NMS delivery performance will significantly expand its development and bring huge value for the design of novel drug delivery systems.

## Figures and Tables

**Table 1:** Detailed textural parameters by N_2_ adsorption measurements.

Sample	Surface Area (cm^2^/g)	Pore Volume (cm^3^/g)	Pore Diameter (nm)
CMSN	440.666	0.358	2.2
NMS/CMSN	9.408	0.043	/
E-CMSN	474.360	1.103	4.3
NMS/E-CMSN	14.635	0.082	/

**Table 2:** The pharmacokinetic parameters of NMS after oral administration of NMS, NMS/CMSN and NMS/E-CMSN (n = 3).

Parameters	NMS	NMS/CMSN	NMS/E-CMSN
AUC_(0–t)_ (mg*h/L)	199.013 ± 66.606	1502.080 ± 217.521	1801.156 ± 134.861
MRT_(0–t)_ (h)	5.729 ± 0.354	10.006 ± 0.157	8.702 ± 0.565
t_1/2z_ (h)	5.081 ± 2.810	5.997 ± 0.107	4.554 ± 0.861
T_max_ (h)	4.000 ± 0.000	6.000 ± 0.000	4.000 ± 0.000
C_max_ (mg/L)	25.442 ± 9.682	100.457 ± 4.939	139.706 ± 9.556
